# Reframing Aging: Penn’s Village Members Thrive While Aging in Place

**DOI:** 10.1093/geroni/igaa057.646

**Published:** 2020-12-16

**Authors:** Lois Evans, Jane Eleey, Avalie Saperstein

**Affiliations:** 1 University of Pennsylvania School of Nursing, Philadelphia, Pennsylvania, United States; 2 Penn’s Village, Philadelphia, Pennsylvania, United States; 3 Penn’s Village, Meadowbrook, Pennsylvania, United States

## Abstract

Villages help older neighbors age-in-place as they manage their environments, take advantage of opportunities for social and civic engagement, and improve or maintain health and well-being. National surveys repeatedly indicate that older adults prefer community living as long as possible. But communities change, and post-retirement living may require rebuilding social connections with old and new neighbors. Fortunately, today’s retirees bring a wealth of knowledge and skills to later life which they are happy to share. Drawing on the talents and career experiences of older adults in Center City, Philadelphia, Penn’s Village (PV) was created in 2007 to address the needs and wishes of neighbors wanting to stay in their own homes as they aged. A member of the Village-to-Village Network, PV (a 501c3) has itself matured through board development, strategic planning, and member engagement. In reframing aging in Center City, PV currently offers its over 300 members and volunteers an array of educational and recreational programs (e.g., talks, affinity groups, social events and outings); services (including transportation, home repairs, IT support, companionship, accompaniment to medical appointments), and opportunities to use their personal knowledge and skills to help their neighbors-- as drivers, companions, volunteer staff and co-chairs/members of committees (e.g., Board of Directors, Program, Welcoming, Marketing & Communications, Finance, Fundraising). In FY2019, volunteers provided nearly 1200 services to PV members. Our most recent survey responses indicate that 82% of volunteers found their work highly meaningful and 79% of those who received services believed their quality of life was greatly improved.

